# Effects of a nurse-led transitional burns rehabilitation programme (4Cs-TBuRP) for adult burn survivors: protocol for a randomised controlled trial

**DOI:** 10.1186/s13063-021-05679-7

**Published:** 2021-10-13

**Authors:** Jonathan Bayuo, Frances Kam Yuet Wong, Loretta Yuet Foon Chung

**Affiliations:** 1 School of Nursing, The Hong Kong Polytechnic University, Hong Kong SAR, China; 2 Evidence-Based Nursing Center, School of Nursing, Lanzhou University, Lanzhou, China

## Abstract

**Background:**

Transitioning from the burn unit to the home/community can be chaotic with limited professional support. Some adult burn survivors may face varied concerns leading to poor outcomes in the early post-discharge period with limited access to professional help. Based on these, a nurse-led transitional burns rehabilitation programme has been developed and the current trial aims to ascertain its effects as well as explore the implementation process.

**Methods:**

A single-centre, double-arm randomised controlled trial with a process evaluation phase will be utilised for this study. All adult burn survivors aged ≥ 18 years with burn size ≥ 10% total burn surface area at the site during the study period will be screened for eligibility at least 72 h to discharge. A sample size of 150 will be block randomised to treatment (receiving the nurse-led transitional care programme and routine post-discharge service) and control groups (receiving routine post-discharge service). The nurse-led transitional care programme comprises of predischarge and follow-up phases with the delivery of bundle of holistic interventions lasting for 8 weeks. There are three timelines for data collection: baseline, immediate post intervention, and 4 weeks post-intervention.

**Discussion:**

The findings from this study can potentially inform the development and organisation of post-discharge care and affirm the need for ongoing comprehensive home-based care for burn survivors and their families

**Trial registration:**

ClinicalTrials.govNCT04517721. Registered on 20 August 2020

## Background and rationale

Burns are one of the most devastating forms of injuries that primarily affect the skin [1]. Depending on the extent of the injury, a burned patient may require critical care to resolve physiological alterations. As care progresses during emergent/acute phase, rehabilitation that occurs concurrently can promote recovery [2–4]. Rehabilitation minimizes the adverse consequences of the burn by improving functional ability, enhancing psychological well-being, facilitating early return to work/school and improving quality of life [5]. As rehabilitation occurs, the burn patient is able to attain a degree of pre-injury abilities as closely as possible [6, 7] and is able to do things that are desirable to them as recover progresses [8, 9].

Along the recovery trajectory for burn survivors, previous studies have observed that the greatest physical and psychosocial decline may be experienced following discharge [10–12]. Concerns regarding pain, anxiety, and uncertainties about return to work/community integration may emerge immediately following discharge [13, 14]. By 2–3 months post-discharge, burn survivors may experience increasing depression/anxiety levels, itchiness associated with healed wounds, pain, poor sleep, scarring, and difficulties with physical functioning/performance leading to poor quality of life in the absence of rehabilitative support commensurate to their needs [13, 15, 16]. By 6–12 months post-discharge, the burn survivor may experience further distress reflected by the high rates of depression, sleep disturbances, body image concerns, and posttraumatic stress disorder [16–18]. Taken together, these may suggest that the transitional period from the immediate pre-discharge to the early post-discharge phase represent a vulnerable stage which can impact long-term recovery outcome [14].

Despite the emergence of varied needs following discharge, there is often limited communication, lack of coordinated and continuous professional support as the burn patient transits from hospital to the home/community [13, 14, 19]. Home care and support may become chaotic, often with limited access to professional support. Some burn survivors may not also return to the hospital for follow-up care due to long travel distances and associated costs. Comprehensive follow-up system of care in the home/community is therefore greatly needed to promote rehabilitation of burn survivors [20]. It is worth mentioning that the home/community has been identified as a suitable location for the implementation of burn rehabilitation programmes which can enhance patient outcomes, particularly if the programme is structured and have active follow-ups on the burn survivor [21].

**Fig. 1:**
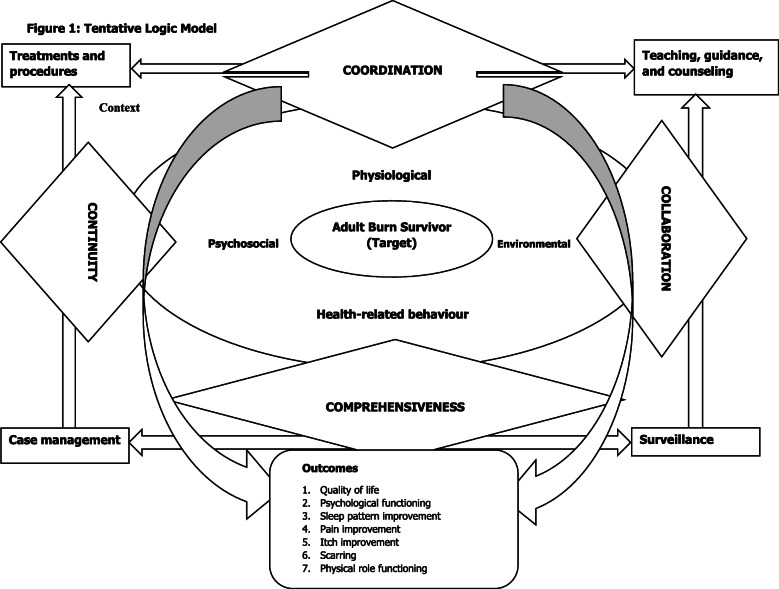
Tentative logic model

In Mainland China, the concept of burns rehabilitation is new to some provinces [22], and only in recent years that rehabilitation of the burn patient was considered an integral part in the Chinese burn care system [23]. Where available, it has been observed that significant challenges exist which include lack of hospital support with no standardised rehabilitation guidelines, inadequate rehabilitation knowledge among practitioners, human resource shortages, limited funding, and financial burden on the burn patients [24]. The rehabilitation protocols, if available, usually focus primarily on the physical aspects of the injury which leaves limited room to attend to the psychosocial concerns of the patient recovering from burns [25]. Consequently, burn patients may not have adequate rehabilitative care as inpatients and with limited support following discharge which adversely affects their recovery process. Thus, to bridge the immediate pre-discharge and early post-discharge gap aiming to improve patient outcomes, a nurse-led transitional burns rehabilitation programme (4Cs-TBuRP) has been developed following the Medical Research Council (MRC) Framework [26] (see Fig. 1, a tentative logic model underpinning the intervention).

Developing the intervention proceeded through extensive literature reviews [27–29], exploratory studies, and expert consultations. The current phase seeks to evaluate the effectiveness of the programme in comparison to the existing standard of care. The 4Cs in the intervention represent comprehensiveness, coordination, collaboration, and continuity, which were proven to be essential transitional care elements tested among patients with chronic conditions [30–32]. The 4Cs emphasise on the need for ongoing holistic care required by burn survivors throughout the recovery trajectory. We ask if the nurse-led transitional rehabilitation programme underpinned by multi-disciplinary support can improve quality of life of burn patients, the primary outcome, in the first 12 weeks following discharge. As secondary outcomes, we will investigate the effects on psychological functioning, sleep patterns, itchiness, pain, and physical role functioning. This study protocol was completed in accordance with the SPIRIT 2013 guidelines [33].

## Objectives

The trial will explore the effects and implementation process of a nurse-led transitional rehabilitation programme for adult burn survivors. We seek to evaluate the effects of a nurse-led transitional rehabilitation programme on the following outcomes among adult burn survivors:

1. Quality-of-life (primary outcome)
2. Psychological functioning
3. Sleep patterns
4. Itchiness
5. Pain
6. Physical role functioning/performance

A secondary objective of this study is to examine the process involved and issues encountered during the implementation of the transitional rehabilitation programme.

## Trial design

The trial is a double-arm (intervention and control groups), single-centre randomised controlled trial with 1:1 allocation ratio and a follow-up process evaluation phase. We will firstly undertake a pilot feasibility phase and follow-up with the full-scale trial. The null hypothesis is that there will be no difference regarding outcomes between the intervention and control groups. The alternative hypothesis is that there will be statistically significant differences between the participants undergoing the nurse-led programme of care and participants receiving the existing standard of care.

The follow-up process evaluation phase will employ a multi-method approach (qualitative and quantitative strands) to examine the process and issues during the implementation process [34]. Twelve process measures covering domains such as recruitment, follow-up, intervention delivery, and data collection will be considered in the process evaluation phase (see Table 1). The qualitative strand will capture changes in the implementation process and experiences with the intervention from the perspectives of adult burn survivors and nurse case managers. The quantitative strand will offer insight into key process variables such as recruitment, follow-up, and adherence rates. Details regarding the process evaluation phase (process measures, variables, methods, and participants) are provided in Table 1.

**Table 1:** Outline of process evaluation for the nurse-led transitional rehabilitation program

Process measures	Process variables	Method	Participants and data collection method
Recruitment and selection rate	• Number of burn survivors screened at the study setting. • Number of participants from the sample of eligible persons. • Number of participants versus aimed number	Enrolment record review	Site manager (research team member)/data collection team
Barriers and facilitators in recruitment and selection process	• Motivation of participating eligible persons. • Experience with recruitment and selection	Semi-structured interviews	Adult burn survivors Recruiting nurses
Follow-up and attrition rate	• Number of participants completing follow-up	Follow-up record review	Site manager (research team member)/data collection team
Barriers and facilitators for follow-up	• Reasons for drop-out • Motivation for continued participation	Semi-structured interviews	Adult burn survivors
Quality of delivery of the interventional components	• The part of each component and the complete intervention delivered by nurse case managers • Satisfaction with delivery	Semi-structured interviews	Nurse case managers
Barriers and facilitators for delivery of interventional components	• Reasons for deviating from, or applying components as planned	Semi-structured interviews	Nurse case managers
Adherence to interventional components	• Number of sessions followed • Intervention components fully implemented and rationale • Intervention components partly implemented and rationale • Intervention components not implemented and rationale • Compliance to individual recommendations	Semi-structured interviews	Adult burn survivors and nurse case managers
Barriers and facilitators for adherence to interventional components	• Motivation for (or lack of) compliance	Semi-structured interviews	Adult burn survivors and nurse case managers
Experience of participants and nurse case managers with the components of the nurse-led interventions and training programme for the nurses.	• Perceived benefit v actual benefit • Strong and weak aspects of the interventional components (structure and content) • Experiences with the educational component (adequacy in assisting burn care nurses to deliver the intervention)	Semi-structured interviews	Adult burn survivors (in intervention group) and nurse case managers
Experience of participants with clinical outcomes associated with the intervention.		Semi-structured interviews	Adult burn survivors (in intervention group)
Completeness of data collection	• Reasons why data were missing • Reasons why participants were excluded from analysis	Semi-structured interviews	Data collection team
Barriers and facilitators for data collection		Semi-structured interviews	Data collection team

## Participants, interventions, and outcomes

### Study setting

This single-centre trial will be conducted at the Gansu Provincial Hospital, Lanzhou, People’s Republic of China. The hospital is a comprehensive top-ranked 3A facility integrating medical services with teaching, research, and preventive care. The facility was established in 1950 and is in the Northwestern part of China. The hospital currently has a total of 3370 beds across various medical and surgical specialties with outpatient facilities.

### Eligibility criteria

All burn survivors receiving burn care services at the study site will be screened for eligibility during the study period. Adult burn survivors aged ≥ 18 years with burn size ≥ 10% TBSA as assessed by the burn surgeon/burn care nurse (irrespective of the depth of the burn), absence of a confirmed psychiatry condition, renal failure, or diabetes mellitus and reachable on phone and WeChat will be considered for inclusion. Adult burn survivors who are unable to communicate or are enrolled in another rehabilitation trial will be excluded. Drop out will be ascertained based on participants exiting either the control or intervention before the 12th week with no intention to continue their participation. Participants for the follow-up process evaluation phase will be recruited from both arms of the trial. The intervention will be primarily provided and coordinated by two burn care nurses with referrals to other members of the multi-disciplinary burns team which comprises of burn surgeons, rehabilitation medicine specialist, and social worker/psychologist who are involved in caring for burn patients at the study site. These burn care nurses recruited to deliver the intervention will have at least 4 years working experience in the burn unit at the study site and must complete a mandatory training programme which covers contents of the comprehensive transitional care program for burn patients with theory and practice (18 h in total) and passed a competence test prior to participating in the study. Other nurses working in the same unit provide routine care to all patients and are not involved in the intervention. Since most of the nurses working in the unit are not involved with the intervention cases, the risk of contamination is minimal.

The objectives of the mandatory training programme were to enable the burn care nurses to be able to master the concept of transitional care; assess, manage, and monitor the transitional needs of burn patients using the Omaha System and make referral when applicable and demonstrate burns rehabilitation competencies (comprehensive scar, itch, and pain management; psychosocial care; aromatherapy; exercises and splinting; monitoring nutrition and hydration; and infection prevention). See Table 2 for details regarding the training programme.

**Table 2:** Details of training programme for burn care nurses based on the TIDieR checklist

TIDieR checklist					
Why (goal/aim, rationale, and theory)	What (materials and procedures), when (schedule), how much (frequency, dose, and intensity of interventions), who (provider), and how (mode of delivery)	Where (location of the intervention)	Tailoring (personalized, titrated or adapted for individual circumstances)	Modifications (changes that occurred during study)	How well (intervention adherence)
The objectives of the mandatory training programme were to enable the burn care nurses to be able to describe transitional care; assess, manage, and monitor the transitional needs of burn patients using the Omaha System and refer when applicable; and demonstrate burns rehabilitation competencies (scar, itch, and pain management; psychosocial care; aromatherapy; exercises and splinting; monitoring nutrition and hydration; and infection prevention).	**What (materials and procedures)** • Overview of transitional care • Overview of the Omaha System and its application to clinical practice • Overview of the rehabilitation program • Comprehensive scar management • Rehabilitation therapies/strategies I: (Psychosocial Care- the STEPS Approach, 3-2-1 GO Strategy, and Counseling). • Positive reinforcement • Meditation • Aromatherapy • Rehabilitation therapies/strategies II: (itch management, pain management, and skin/wound care). • Exercises/splinting/positioning • Nutrition-hydration • Infection precautions • Undertaking referrals **When (schedule)** • Training scheduled from 28th August 2020 to 2nd September 2020. • Personal revision schedule **How much (frequency and dose)** • 4.5 h per session (18 h in total) **Who (Provider)** • One professor of rehabilitation • One professor of nursing • One burn care nurse/doctoral student **How (mode of delivery)** • Online via Zoom	Gansu Provincial Hospital	Though the training programme was designed for the group, the facilitators offered assistance to individuals based on questions raised	No modifications made	Entire training schedule was duly adhered to with no deviation

### Intervention

Eligible participants will be randomised to either the treatment or control group. Participants in the control group will continue to utilise optimised usual are comprising of the existing service at the hospital in addition to two social calls by a trained student nurse. Participants in the treatment group will receive the nurse-led transitional burns rehabilitation programme (4Cs-TBuRP) in addition to existing service at the hospital. There are two phases to the 4Cs-TBRP:

- Phase 1 (discharge planning/preparation): This phase will take place at least 72 h prior to the day of discharge. Guided by the Omaha System, a comprehensive patient assessment will be performed by the assigned burn care nurse based on a care plan covering environmental, physiological, psychosocial, and health-related behaviour domains will be carried out [35]. The bundle of interventions available is based on the identified need which include environmental (health education to improve sanitation and environmental hygiene within residence), physiological (pain management, itch management, comprehensive scar management, range of motion activities, management of heat sensitivity, skin care, nutritional assessment/support, infection prevention and control measures), psychosocial (social skills training, counselling care, health education, positive reinforcement, spiritual care, meditation, and music therapy), and health-related behaviour (strategies to improve sleep/rest, physical therapy, and participation in activities of daily living). See Table 3 for details regarding the bundle of intervention presented based on the TIDieR checklist.
- Phase 2 (follow-up phase): This phase comprises of 2 structured WeChat telehealth, 6 structured telephone follow-ups and daytime patient/family-initiated telephone service over an 8-week period. Like the discharge planning phase, care planning and intervention are undertaken using the Omaha System as a guide for the 8-week period. The bundle of interventions available based on the identified need/domain include environmental (health education to improve sanitation and environmental hygiene within residence), physiological (pain management, itch management, comprehensive scar management, range of motion activities, management of heat sensitivity, skin care, nutritional assessment/support, infection prevention and control measures), psychosocial (social skills training, counselling care, health education, positive reinforcement, spiritual care, meditation, music therapy and aromatherapy), and health-related behaviour (strategies to improve sleep/rest, physical therapy, and participation in activities of daily living). Where referrals are required, a referral form will be completed for the service to be provided. Home visits will be carried out if the adult burn survivor has concerns which can best be handled face to face by the nurse (example: assistance with wound care or reinforcing scar management education). See Table 3 for details regarding the bundle of intervention presented based on the TIDieR checklist.

**Table 3:** Bundle of intervention

Phase of intervention	TIDieR checklist					
	What (procedures)	Who	How (mode of delivery)	Where (location of the intervention)	When (schedule), and How much (frequency, dose and intensity of interventions)	Tailoring (personalized, titrated or adapted for individual circumstances)
**Discharge planning phase (at least 72 h to discharge)**	Comprehensive patient assessment using the OMAHA System within one week for an adult burn survivor who has a pending discharge status. Interventions will depend on the identified needs and should be classified into teaching, guidance and counselling, treatment and procedures, case management and surveillance. Please ensure to use the intervention scheme for each need. For needs where corresponding schemes are not available, please use the ISBI guidelines.	Nurse case manager (holds a bachelor’s degree or in nursing, and a minimum of 4 years working experience in the Burn Unit)	Face to face and with each patient individually	Burn Unit of the hospital	As needs may change, the first assessment serves as a reference point. The adult burn survivor should be assessed prior to discharge by the nurse case manager.	All components of the intervention scheme may not apply at the same time. Please ensure to match needs at a time to the specific intervention
	Providing support	Nurse case manager	Face to face and with each patient individually	Conducted in the Burn Unit	Once before discharge for 30–45 min (the discussion will be recorded for monitoring and evaluation purposes)	The adult burn survivor is permitted to ask questions beyond the scope of the guidelines; however, the standard components should be covered entirely
**Day of discharge**	Reminder about WeChat Telehealth Service, daytime patient/family-initiated call options and follow-up by the Nurse Case Manager; follow-up on previously identified problems during discharge planning phase	Nurse case manager	Face to face with each patient	Burn Unit	Day of discharge	The assessment is the same, but interventions will vary depending on the goals set with the patient
**Follow-up phase**	Follow-up call for the following: • To confirm home address • Remind the patient/family caregiver of the availability of 24-h hotline service • Follow-up on previously established goals	Nurse case manager	On phone	Home	Phone call 24 h after discharge once (please note for quality and evaluation purposes, the discussion over the phone will be monitored and recorded)	
24 h after discharge						
One-week post-discharge	First structured telephone chat (comprehensive patient assessment should be completed, and interventions instituted where necessary)	Nurse case manager	Via telephone	On phone	Once	Emerging needs may differ, and interventions should be aligned to the needs that are identified
2nd week post-discharge	First WeChat Telehealth follow-up	Nurse case manager	Via WeChat			
3rd week post-discharge	Second structured telephone follow-up	Nurse case manager	On phone	phone	Once	Patients may activate the 24-h service as their needs/concerns evolve (all discussions will be recorded)
4th week post-discharge	Third structured telephone follow-up	Nurse case manager	Phone	phone	Once	Patient’s goals may change or remain same; needs may vary, and interventions should match the identified needs
5th week post-discharge	Fourth structured telephone follow-up	Nurse case manager	On phone	Phone	Once	Patients may activate the 24-h service as their needs/concerns evolve (all discussions will be recorded)
6th week post-discharge	Fifth structured telephone follow-up	Nurse case manager	On phone	Phone	Once	Patients may activate the 24-h service as their needs/concerns evolve (all discussions will be recorded)
7th week post-discharge	Sixth structured telephone follow-up	Nurse case manager	On phone	Phone	Once	Patients may activate the 24-h service as their needs/concerns evolve (all discussions will be recorded)
8th week post-discharge	Second WeChat follow-up	Nurse case manager	Virtual	Virtual	Once	Emerging needs may differ, and care should be aligned to the needs that are identified

### Strategies to improve adherence/fidelity of the intervention

Fidelity will be ensured in this study by using the guidelines offered by Bellg et al. [36] which comprises of (1) ensuring that the intervention dose is the same for all participants across each condition, (2) standardising interventionist training, (3) monitoring the intervention delivery, (4) evaluating participants’ understanding of information provided, and (5) ensuring that participants use the skills taught in the intervention. Adherence reminders will also be given every week to participants during the follow-up via WeChat or telephone. Burn care nurses will document date and length of contacts per participant, referrals to specialists, and performance of intervention components. All calls will be documented, and WeChat messages/images will be reviewed by the team on monthly basis. Also, the care plan sheets for each participant will be reviewed by the research and clinical team via case conferencing held bi-monthly to discuss assessment, management, and evaluation in relation to the study protocol. Nurse case managers will be encouraged to maintain a clinical diary which will be reviewed.

### Study outcomes

#### Primary outcome

*Quality of life*: Quality of life which is defined as the adult burn survivors’ perceptions of their health status and well-being is the primary outcome for the trial and will be assessed at baseline (T0), following completion of the intervention at 8 weeks (T1) and at 4 weeks post-intervention (T2). The outcome will be objectively assessed using the Chinese version of the Burn Specific Health Scale-Brief and EQ-5D-5L. The BSHS is an outcome scale designed specifically for burns patients. It comprises of four areas: physical, psychological, social relations, and general health condition that is consistent with the biopsychosocial model. A recent review has suggested that generic scales are more sensitive than the BSHS-B domains from one-month post-burn [37]. Thus, as the study will cover up to 3 months post-discharge, the BSHS-B and EQ-5D will be used to measure quality of life in the study. The BSHS-B comprises of 40 questions, 9 sub-scales, and 3 domains (physical, mental, and social). Internal consistency of the total instrument has been reported to be *α* = 0.94 with test-retest reliability suggesting that the intraclass correlation coefficient (ICC) ranged from 0.81 to 0.96 with a total score of 0.93 [38]. The Chinese version has 38 items with a reported Cronbach alpha 0.97 and split half reliability of 0.98 [39]. The EQ-5D-5L is a generic tool to assess quality of life. The instrument has five dimensions: mobility, self-care, usual activities, pain/discomfort, and anxiety/depression. Each dimension has 5 levels: no problems, slight problems, moderate problems, severe problems, and extreme problems. The EQ-5D-5L is a valid extension of the 3-level system (EQ-5D-3L) which is found to have improved measurement properties, with reduced ceiling effect, strengthened discriminatory power and established convergent and known-groups validity [40].

#### Secondary outcomes

Assessment of secondary outcomes will also commence at baseline T0 (recruitment to the study), following completion of the intervention at 8 weeks (T1) and at 4 weeks post-intervention (T2):

*Psychological functioning*: The Chinese version of the Hospital Anxiety and Depression Scale (HADS) will be used to evaluate psychological functioning (anxiety and depression). The 14-item *HADS* was designed as a tool to screen for anxiety and depressive symptoms in medical settings and has well-demonstrated solid psychometric properties for use in medical settings [41–43] and commonly used in burn care settings to screen anxiety and depression [44].

*Sleep pattern improvement*: The Chinese version of the Pittsburgh Sleep Quality Index (PSQI) will be used to evaluate sleep improvement. It includes 19 items with a total score that ranges from 0 to 21. A higher PSQI score implies poorer quality of sleep [45]. In general, a PSQI score greater than 5 is considered to indicate poor sleep quality [45].

*Pain*: The Chinese version of the Brief Pain Inventory will be used to evaluate pain among participants. It has 15 items, of which 11 items have numeric rating scale, on the experience of pain, the area of severe pain, the presence of pain, minimum and maximum pain on the previous day, current pain level, analgesia and pain relief, and the interference of pain on the patient’s functioning [46].

*Itch*: The Chinese version of a visual analogue scale (VAS) will be used to evaluate this outcome among participants. The VAS has been observed to have an intraclass correlation coefficient of 0.88 [47].

*Physical role functioning*/*performance*: The Chinese version of the Disability of the Arm, Shoulder and Hand Symptom Scale (DASH) will be used to evaluate this outcome [48]. The DASH tool is a 30-item questionnaire that asks the patient to grade symptoms and physical function during the preceding week on a five-point Likert scale. The reliability of the DASH is excellent (intraclass correlation coefficient 0.97 with Cronbach’s alpha of 0.97) [48].

*Scarring*: The Vancouver Scar Scale (VSS) will be used to assess this outcome in the current study. The VSS is a validated subjective tool that assesses vascularity, pigmentation, pliability, and height of scars [49]. The tool remains the main tool used to evaluate scarring in burn care research [49]. The total score ranges from 0 to 13 with lower scores implying improved scars.

### Participant timeline

**Fig. 2:**
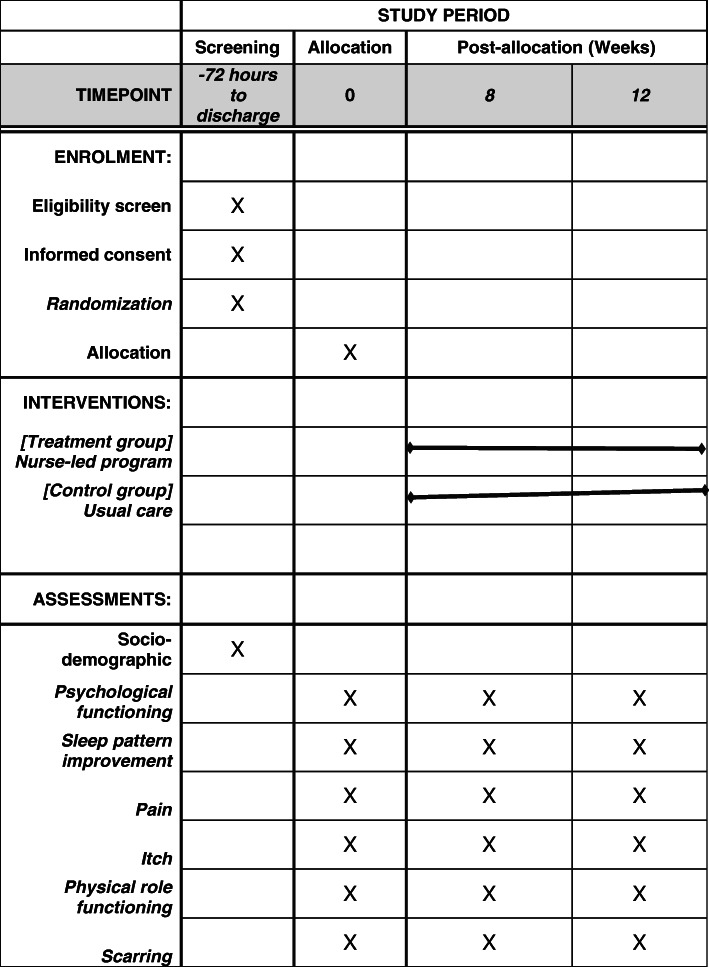
Schedule of enrolment, interventions, and assessments for the nurse-led transitional rehabilitation programme

All burn survivors at the study site will be screened for eligibility. Enrolment to the study will take place 72 h to discharge from the burn unit. Baseline data collection with a battery of outcome measures along with demographic data. The intervention commences for participants in the treatment group with the pre-discharge component of the programme of care. Weekly follow-ups will be conducted by the nurse case managers. The second phase of data collection will be completed by 8 weeks post-intervention. Follow-up data will be obtained at 4 weeks following the second data collection phase (see Fig. 2).

### Sampling and sample size estimation

Subjects will include adult burn survivors with burn size ≥ 10% TBSA from the Burn Unit. Upon recruitment, randomisation to either control or treatment group (ratio 1:1) with a blinded approach will be carried out. For the pilot phase of the project, the pilot feasibility sample size rule suggested by Whitehead, Julius, Cooper, & Campbell will be used [50]. The rule specifies that a total sample size of 25 participants per arm. Considering possible attrition rate of 20% that has been reported in burn care literature implies that an additional 5 participants will be added to both arms summing up to 30 participants per arm (total = 60 participants).

For the main trial, the sample size calculation based on the primary outcome measure (Burn Specific Health Scale) at 95% confidence level, 90% statistical power and moderate effect size will yield a total of 38 participants per arm [51–53]. Considering an attrition rate of 20%, the final sample size will be 45 participants per arm (total = 90 participants). In all, 150 participants will be recruited for the entire study. For the follow-up process evaluation phase, we will conveniently recruit up to fifteen [15] participants from each arm whilst being guided by the principle of data saturation in addition to the burn care nurses delivering the intervention to complete the qualitative strand. Data saturation will be determined at the point where no new data emerges. The quantitative strand will include review of the recruitment, enrolment, and follow-up records.

### Recruitment

**Fig. 3:**
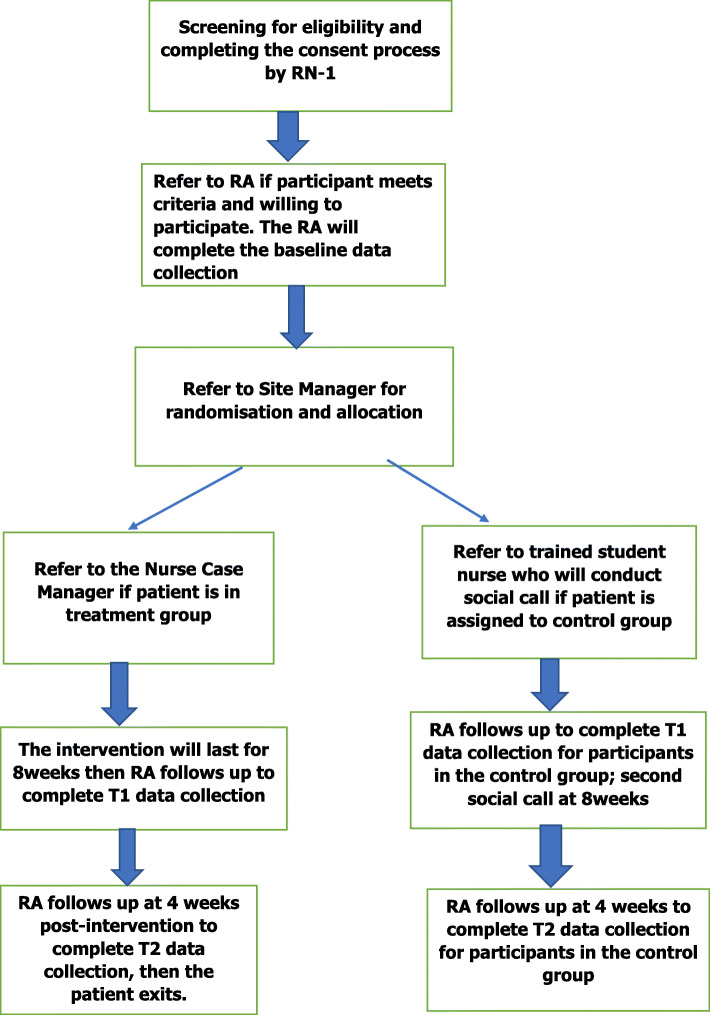
Work flowchart

A research nurse at the study site will identify potential subjects’ eligibility for enrolment in the study based on the inclusion and exclusion criteria described above. All participants will be enrolled/recruited by a Registered Nurse (RN-1) who will not be involved in the delivery of the intervention or outcome assessment (see Fig. 3). RN-1 will follow-up on patient’s case notes on daily basis to ascertain if any adult burn survivor has a pending discharge status. If pending discharge is noted, RN-1 will check if the adult burn survivor fulfils the criteria for inclusion; if so, the patient will be invited to participate. Details of the study will be made known and information sheet duly explained. Thereafter, RN-1 will ensure that two consent forms (covering both outcome and process evaluation phases) are signed or thumb printed by the adult burn survivor which will then be forwarded to a research assistant (RA) who will complete the baseline assessment. A research team member (RTM) who is not involved in clinical services and have no knowledge about the potential subjects will take over from this phase and proceed to work on randomisation and allocation. The subjects allocated to the intervention arm will be referred to the nurse case manager to commence the intervention. Following the exit of participants from the intervention, they will be contacted and invited to participate in the process evaluation phase.

## Assignment of interventions

### Sequence generation, randomisation, and allocation

Eligible participants who provide written, informed consent will be randomised on a 1:1 basis to one of two trial arms. Prior blocked randomisation list with 75 sets of numbers will be generated using the Research Randomizer and kept in sealed envelopes. As soon as the consent form is received, the RTM will sequentially open the sealed envelopes containing the paper with the number assignment and unfold it. The number written specifies the group to which the adult burn survivor will be allocated. The allocation sequence will be concealed to RN-1 and the research assistant (RA) to minimise bias in the study.

### Implementation

The study team will enrol participants face to face from the burn unit of the Gansu Provincial Hospital. The doctoral student will generate the blocked randomisation list and forward it to the site manager who will then place each list in an envelope and seal them afterwards. These will be passed on to the research team member working on randomisation.

### Blinding

Following recruitment, any possible identifying material that could indicate to the blinded assessors, which group a participant was allocated to, will be removed. Participants will not be blinded as to the group they have been assigned to. Additionally, it will not be possible to blind the interventionists, that is, the burn care nurses who will deliver the intervention only to participants in the treatment group.

## Data collection, management, and analysis

### Data collection methods and management

All outcomes will be assessed at baseline (T0); that is after the patient has been recruited. Following the completion of the intervention, the second phase of data collection (T1) will be undertaken; that is at the 8th week. The third phase of data collection (T2) will be the follow-up phase at 4 weeks post-intervention. Data collection will be in the form of completion of questionnaires. The data collection process will be undertaken by three research assistants who will be blinded to the groups of the participants. The research assistants have completed a 10-h training programme covering the outcome measures to be used. Following the training, they undertook a role play to enable the research team to ascertain the inter-rater reliability/percentage agreement with the following results: Burn Specific Health Scale-B (87.7%), EQ-5D-5L (100%), HADS (92.5%), PSQI (86.7%), DASH (84.4%), VAS (100%), BPI (100%), and VSS (100%). All quantitative data will be entered and stored in SPSS Version 25 (SPSS, an IBM company, Chicago, Illinois, USA). All hard copies of documents relating to the study will be stored under lock and key only accessible to designated research team member.

For the follow-up process evaluation phase (qualitative strand), face to face semi-structured interviews with an interview guide will be utilised to collect data after the intervention period has elapsed. Topics for the interview guide will be based on the process evaluation components as highlighted in Table 1. All interview proceedings will be audiotaped and transcribed verbatim in the Trint Automated Transcription Software. Interview transcripts will be exported to QSR NVivo version 10 will be used to manage the data. All soft files related to the study will be kept in a password protected computer only accessible to the principal investigator and the doctoral student investigator. Paper files will be kept under lock and key. For the quantitative phase, we will review the recruitment, enrolment, and follow-up records.

### Statistical methods and analysis

The data analysis plan will proceed through data entry and cleaning, missing data management and statistical analysis. All quantitative data will be coded and entered to SPSS Version 25 (SPSS, an IBM company, Chicago, Illinois, USA). The data will be inspected visually with random searches and frequency counts to ascertain its completeness. Demographic and clinical characteristics will be compared using chi-square test (for normally distributed data) or Mann-Whitney *U* test (data that is not normally distributed). Between group differences will be assessed using independent t test (for continuous data). Both per-protocol (PP) and intention to treat analysis (ITT) will be performed respectively on participants who completed the entire intervention and those recruited to participate. Methods for longitudinal data such as generalised estimating equations (GEE) will be employed. All statistical tests will be conducted at 95% confidence interval.

Qualitative data emerging from the follow-up process evaluation phase will be analysed using content analysis [54]. All audio recordings will be transcribed verbatim and translated from Chinese to English using the Trint Automation Software. The transcripts will be inspected for correctness and completeness by two native speakers who are also fluent in English. The final versions will be exported to the NVivo version 10 software for data management and analysis by two researchers with ongoing consultation with the research team. The analytical process will commence with reading all data repeatedly to achieve immersion and obtain a sense of the whole. Then, data will be read line by line to formulate codes by first highlighting the exact words from the text that appear to capture key thoughts. Following this, the researcher will approach each transcript by making notes of initial impressions. Following the formulation of the codes, labels for the code will be formulated directly from the text. At this point, an initial coding scheme will be formulated. Similar codes will be sorted to formulated categories. Emergent categories will be used to organize and group codes into clusters [54].

Data emerging from the quantitative strand will collated into SPSS Version 25. We will employ descriptive statistics to ascertain the distribution of the data.

## Monitoring

Minimal adverse effects are expected from the proposed intervention. Standardised protocols exist at the study centre regarding the management of known of potential adverse effects such as infection. Adverse events related to the intervention will be monitored through review of the patient’s records, participant self-report, and by the clinicians. All adverse events will be reported to the Institutional Review Board of the Hong Kong Polytechnic University.

### Ethics and dissemination

Ethical clearance for the study has been granted by the Institutional Review Board of the Hong Kong Polytechnic University (HSEARS20200730001). Additionally, the study has been prospectively registered with a trial registry and currently available in public domain (NCT04517721). The IRB and trial registry will be informed of any modifications that may occur during the conduct of the trial. Protocol modifications which affect participants directly after enrolling in the trial will be communicated directly to participants via the research team member. Consent will be obtained from participants before completing the recruitment process. All data emerging from the studies will be kept under lock kept by authorised personnel. Confidentiality and anonymity will be ensured throughout the study. Data emerging from the study will be accessed only by the research and clinical teams. The protocol for this study is publicly available. Additional details will be provided upon reasonable request. Statistical codes for analysis will be published with the final publication of the trial results when completed. De-identified participant-level data may be made available upon reasonable request, after reviewing the request by the study team and the local institution. Trial auditing will be conducted every six weeks by the research and clinical team to ensure adherence/compliance to the protocol. The investigators plan to disseminate the trial results via publication in peer reviewed journals and at conferences. All research and clinical team members meeting authorship criteria will have final authority over manuscript content. The study protocol, if published, will be available in public domain. However, participant-level dataset will be maintained by the research/team which can be made available on reasonable request.

### Ancillary and post-trial care

This is not applicable. No specific provisions for post-trial care were considered in this trial due to the nature of the intervention. All harms and adverse events (AE) will be dealt properly, and if the participants need any health care due to participation in the study, they will be referred to the respective referral services.

## Discussion

Recovery following burns is a complex process with varied biopsychosocial-environmental needs and rehabilitative support commensurate to those needs is very important to ensure optimal recovery of the burn survivors [55]. Recent studies have likened the recovery process following burns to living with a chronic ailment which suggest that a flexible but structured and sustained follow-up plan is required to support the burn survivor and their families [56, 57]. Transitioning from the Burn Unit to the home/community is often chaotic with burn survivors and their families experiencing limited professional support [57, 58]. A lack of continuous and comprehensive support in the early post-discharge period adversely affects patient outcomes, thus creating a critical gap.

The concept of rehabilitation is still under development in most parts of Mainland China [4, 24, 59, 60]. The escalating chronic disease burden and ageing population further leaves limited resources allocated to burns rehabilitation in Mainland China [60]. Previous studies in Mainland China have highlighted the significant focus on medical management of burn injuries with limited attention to comprehensive rehabilitative support [4, 24]. Thus, service gaps may exist to offer comprehensive rehabilitative support to burn survivors. Meeting this need requires multidisciplinary efforts to ensure that burn survivors and their families receive professional support commensurate to their needs in a continuous and coordinated manner. The nurse-led transitional burns rehabilitation programme (4Cs-TBuRP) will add new knowledge regarding the organization and implementation of professional support for adult burn survivors and their families as well as strengthening post-discharge care at the study hospital. The programme extends the function of burn care nurses into advanced roles regarding coordinating care and delivering advanced burn care support such as scar management in home-based environment. Additionally, the follow-up process evaluation phase will offer insights into the implementation process to understand contextual factors that facilitated or hindered the delivery of the intervention. By combining quantitative and qualitative data, we will attain greater explanatory power and move beyond ‘does it work?’ to ‘what works and for who?’ Based on past trials, an anticipated limitation of this study may be dropout rate. Possible attrition bias will be addressed by examining the extent and reasons for dropout across the intervention and control groups.

In conclusion, the 4Cs-TBuRP offers an opportunity to actively follow-up on burn survivors and their families following discharge and offer professional support within the home environment. This is in response to a global need for continued rehabilitative care for burn survivors following discharge [14]. The telehealth component which reflects the changing nature of healthcare particularly as the world is still navigating through the coronavirus pandemic implies that burn survivors will continue to receive professional support at a distance highlighting the potential benefits of the 4Cs-TBuRP [61, 62].

## Data Availability

Not applicable
